# Supplementary material for: The Lipid- and Polysaccharide-Rich Extracellular Polymeric Substances of Rhodococcus Support Biofilm Formation and Protection from Toxic Hydrocarbons
Source: Polymers (Basel). 2025 Jul 10;17(14):1912. doi: 10.3390/polym17141912 (PMC12298843; doi:10.3390/polym17141912)
Supplement: Supplementary file 1 [file polymers-17-01912-s001.zip › Krivoruchko_et_al_Supplementary.pdf]

Table S1. *Rhodococcus* strains used in this study

No.	Species	Strains	Isolation source	Link to the IEGM Collection	DDBJ/ ENA/ Genbank acc. no
1	*Rhodococcus aetherivorans*	IEGM 1250	Soil, Russia	http://iegmcol.ru/strains/rhodoc/aether/r_aether1250.html, last accessed 21 May 2025	Whole genome is not sequenced
2		IEGM 1367	Soil, village of Letnyaya Zolotitsa, coast of the White Sea, Arhangelsk region, Russia	http://iegmcol.ru/strains/rhodoc/aether/r_aether1367.html, last accessed 21 May 2025	JAWLKG010000001-JAWLKG010000080
3	*Rhodococcus cerastii*	IEGM 1243	Soil, lake shore Kumnylor, Tyumen region, Russia	http://iegmcol.ru/strains/rhodoc/cerastii/r_cerastii1243.html, last accessed 21 May 2025	JAJNDD010000001-JAJNDD010000295
4		IEGM 1278	Oil-polluted soil after the remediation, Udmurt Republic, Russia	http://iegmcol.ru/strains/rhodoc/cerastii/r_cerastii1278.html, last accessed 21 May 2025	Whole genome is not sequenced
5		IEGM 1327	Bottom sediment, Kosmicheskoye Lake, Hays Island, Franz Josef Land, Arkhangel'sk region, Russia	http://iegmcol.ru/strains/rhodoc/cerastii/r_cerastii1327.html, last accessed 21 May 2025	JAWLKF010000001-JAWLKF010000052
6	*Rhodococcus cercidiphylli*	IEGM 1184	*Chenopodium* rhizosphere, highway to salt dump, Solikamsk, Perm region, Russia	http://iegmcol.ru/strains/rhodoc/cercid/r_cercid1184.html, last accessed 21 May 2025	Whole genome is not sequenced
7	*Rhodococcus corynebacterioides*	IEGM 1202	Coastal soil, the Goltsovoye Lake, the Mammoth Peninsula, Yamalo-Nenets Autonomous Okrug, Tyumen region, Russia	http://iegmcol.ru/strains/rhodoc/corynebac/r_corynebac1202.html, last accessed 21 May 2025	JAWLUV010000001-JAWLUV010000028
8	*Rhodococcus erythropolis*	IEGM 708	Oil-shale from settling pit, Polazna oil-extracting enterprise, Perm region, Russia	http://iegmcol.ru/strains/rhodoc/eryth/r_eryth708.html, last accessed 21 May 2025	JAPWIP010000001-JAPWIP010000169
9		IEGM 766	Oil-polluted soil, oil-extracting enterprise, Udmurt Republic, Russia	http://iegmcol.ru/strains/rhodoc/eryth/r_eryth766.html, last accessed 21 May 2025	JAPWIK010000001-JAPWIK010000097
10		IEGM 788	Oil-polluted soil, Novosibirsk region, Russia	http://iegmcol.ru/strains/rhodoc/eryth/r_eryth788.html, last accessed 21 May 2025	JASHLI010000001-JASHLI010005190
11		IEGM 1020	Water, enterprise on production of paracetamol, Perm region, Russia	http://iegmcol.ru/strains/rhodoc/eryth/r_eryth1020.html, last accessed 21 May 2025	JAWLKD010000001-JAWLKD010000053

12		IEGM 1321	Bottom sediments, stream, Kane Island, Franz Josef Land, Novaya Zemlya, Arkhangel'sk region, Russia	http://iegmcol.ru/strains/rhodoc/eryth/r_eryth1321.html, last accessed 21 May 2025	JAWLKC010000001-JAWLKC010000382
13		IEGM 1348	Cd-contaminated soil, metallurgical enterprise area, Perm, Russia	http://iegmcol.ru/strains/rhodoc/eryth/r_eryth1348.html, last accessed 21 May 2025	JASIRM010000001-JASIRM010000081
14		IEGM 1399	Ground, Antarctica	http://iegmcol.ru/strains/rhodoc/sp/r_sp1399.html, last accessed 21 May 2025	JAPWIH010000001-JAPWIH010000081
15		IEGM 1415	Oil-polluted soil, Norilsk, Krasnoyarsk region, Russia	http://iegmcol.ru/strains/rhodoc/eryth/r_eryth1415.html, last accessed 21 May 2025	JAPWIQ010000001-JAPWIQ010000044
16	*Rhodococcus fascians*	IEGM 1233	*Elytrigia repens* rhizosphere, oilfield, Perm region, Russia	http://iegmcol.ru/strains/rhodoc/fascians/r_fasc1233.html, last accessed 21 May 2025	JASHLH010000001-JASHLH010000075
17	*Rhodococcus globerulus*	IEGM 1203	Soil, lake shore Gal'tsovoye, Tyumen region, Russia	http://iegmcol.ru/strains/rhodoc/glober/r_globerulus1203.html, last accessed 21 May 2025	JAWLKB010000001-JAWLKB010000134
18	*Rhodococcus jostii*	IEGM 60	Oil-polluted soil, oilfield, Ukraine	http://iegmcol.ru/strains/rhodoc/jostii/r_jostii60.html, last accessed 21 May 2025	JAWLKA010000001-JAWLKA010000101
19		IEGM 68	Soil, Polasna oil-extracting enterprise, Perm region, Russia	http://iegmcol.ru/strains/rhodoc/jostii/r_jostii68.html, last accessed 21 May 2025	JAWLUP010000001-JAWLUP010000452
20	*Rhodococcus opacus*	IEGM 249	Soil, lavsan (polyether fibre) production, Belarus	http://iegmcol.ru/strains/rhodoc/opac/r_opac249.html, last accessed 21 May 2025	JAPWIS010000001-JAPWIS010000138
21		IEGM 262	Soil, lavsan (polyether fibre) production, Belarus	http://iegmcol.ru/strains/rhodoc/opac/r_opac262.html, last accessed 21 May 2025	Whole genome is not sequenced
22		IEGM 2226	Oil-polluted soil, Perm region, Russia	http://iegmcol.ru/strains/rhodoc/opac/r_opac2226.html, last accessed 21 May 2025	JAWLJZ010000001-JAWLJZ010000268
23	*Rhodococcus pyridinivorans*	IEGM 66	Oil-polluted soil	http://www.iegmcol.ru/strains/rhodoc/rhodoch/r_rhod66.html, last accessed 21 May 2025	JAJNDE010000001-JAJNDE010000099
24		IEGM 1142	Rhizosphere, Sverdlovsk region, Russia	http://iegmcol.ru/strains/rhodoc/pyridin/r_pyridin1142.html, last accessed 21 May 2025	JAJNCQ010000001-JAJNCQ010000056
25	*Rhodococcus qingshengii*	IEGM 267	Oil-polluted soil, oil-extracting enterprise, Perm region, Russia	http://iegmcol.ru/strains/rhodoc/qingsh/r_qingsh267.html, last accessed 21 May 2025	MRBQ01000001-MRBQ01000231
26		IEGM 1270	Oil slime, Udmurt Republic, Russia	http://iegmcol.ru/strains/rhodoc/qingsh/r_qingsh1270.html, last accessed 21 May 2025	JANFQL010000001-JANFQL010000087
27		IEGM 1359	Bottom sediment from lake systems, Li Smita Island, Franz Josef	http://iegmcol.ru/strains/rhodoc/qingsh/r_qingsh1359.html, last accessed 21 May 2025	JAJNCL010000001-JAJNCL010000048

			Land, Arkhangel'sk region, Russia.		
28	*Rhodococcus rhodochrous*	IEGM 64	No information	http://iegmcol.ru/strains/rhodoc/rhodoch/r_rhod64.html, last accessed 21 May 2025	Whole genome is not sequenced
29		IEGM 107	Water, the Dnieper River, Dnepropetrovsk region, Ukraine	http://iegmcol.ru/strains/rhodoc/rhodoch/r_rhod107.html, last accessed 21 May 2025	JAJNCP010000001-JAJNCP010000118
30		IEGM 1161	*Atriplex* rhizosphere, soil, former landfill area, Perm, Perm region, Russia	http://iegmcol.ru/strains/rhodoc/rhodoch/r_rhod1161.html, last accessed 21 May 2025	JASHLG010000001-JASHLG010000094
31		IEGM 1162	*Atriplex* rhizosphere, soil, former landfill area, Perm, Perm region, Russia	http://iegmcol.ru/strains/rhodoc/rhodoch/r_rhod1162.html, last accessed 21 May 2025	Whole genome is not sequenced
32		IEGM 1298	Water, lake Arantur, Tyumen region, Russia	http://iegmcol.ru/strains/rhodoc/rhodoch/r_rhod1298.html, last accessed 21 May 2025	JAWLUO010000001-JAWLUO010000072
33		IEGM 1360	Moss rhizosphere, soil near a stream at hydropost, bukhta Tikhaya, Gukera Island, Franz Josef Land, Arkhangel'sk region, Russia	http://iegmcol.ru/strains/rhodoc/rhodoch/r_rhod1360.html, last accessed 21 May 2025	JAJNCN010000001-JAJNCN010000105
34		IEGM 1362	Peat, Pal'tinskoye peat deposit, Perm region, Russia	http://iegmcol.ru/strains/rhodoc/rhodoch/r_rhod1362.html, last accessed 21 May 2025	JANFQM010000001-JANFQM010000140
35		IEGM 1363	Oil-polluted soil, Udmurt Republic, Russia	http://iegmcol.ru/strains/rhodoc/rhodoch/r_rhod1363.html, last accessed 21 May 2025	JASIRJ010000001-JASIRJ010000200
36	*Rhodococcus ruber*	IEGM 231	Water, spring, Olkhovski oil-extracting enterprise, Perm region, Russia	http://iegmcol.ru/strains/rhodoc/ruber/r_ruber231.html, last accessed 21 May 2025	CCSD01000001-CCSD01000115
37		IEGM 560	Sand from the depth 6.0 m, Belarus	http://iegmcol.ru/strains/rhodoc/ruber/r_ruber560.html, last accessed 21 May 2025	JASHLB010000001-JASHLB010000163
38		IEGM 1121	*Plantago* rhizosphere, soil, former landfill area, Perm, Perm region, Russia	http://iegmcol.ru/strains/rhodoc/ruber/r_ruber1121.html, last accessed 21 May 2025	JASHLF010000001-JASHLF010000239
39		IEGM 1122	*Plantago major* rhizosphere, soil, former landfill area, Perm, Perm region, Russia.	http://iegmcol.ru/strains/rhodoc/ruber/r_ruber1122.html, last accessed 21 May 2025	JASIRH010000001-JASIRH010000151
40		IEGM 1135	*Poa pratensis* rhizosphere, soil, highway to salt	http://iegmcol.ru/strains/rhodoc/ruber/r_ruber1135.html, last accessed 21 May 2025	Whole genome is not sequenced

			dump, Solikamsk, Perm region, Russia		
41		IEGM 1263	Oil-polluted waste, Sosnogorsk, Komi Republic, Russia	http://iegmcol.ru/strains/rhodoc/ruber/r_ruber1263.html, last accessed 21 May 2025	JAWLJY010000001-JAWLJY010000093
42		IEGM 1391	Oil-polluted soil, Udmurt Republic, Russia	http://iegmcol.ru/strains/rhodoc/ruber/r_ruber1391.html, last accessed 21 May 2025	JAPWIJ010000001-JAPWIJ010000062
43	*Rhodococcus wratislaviensis*	IEGM 1171	*Urtica dioica* rhizosphere, soil, petrol station, Perm, Perm region, Russia	http://iegmcol.ru/strains/rhodoc/wratis/r_wratis1171.html, last accessed 21 May 2025	JAWLUN010000001-JAWLUN010000162
44	*Rhodococcus* sp.	IEGM 1401	ground, Antarctica	http://www.iegmcol.ru/strains/rhodoc/sp/r_sp1401.html, last accessed 21 May 2025	JAPWIM010000001-JAPWIM010000134
45		IEGM 1408	Bottom sediments, Severnaya Zemlya, Krasnoyarsk region, Russia	http://iegmcol.ru/strains/rhodoc/sp/r_sp1408.html, last accessed 21 May 2025	JAWLRW010000001-JAWLRW010000062
46		IEGM 1409	Bottom sediments, Severnaya Zemlya, Krasnoyarsk region, Russia	http://iegmcol.ru/strains/rhodoc/sp/r_sp1409.html, last accessed 21 May 2025	JASHKN010000001-JASHKN010000058
47		IEGM 1414	Water, Severnaya Zemlya, Krasnoyarsk region, Russia	http://iegmcol.ru/strains/rhodoc/sp/r_sp1414.html, last accessed 21 May 2025	JAWLRV010000001-JAWLRV010000128

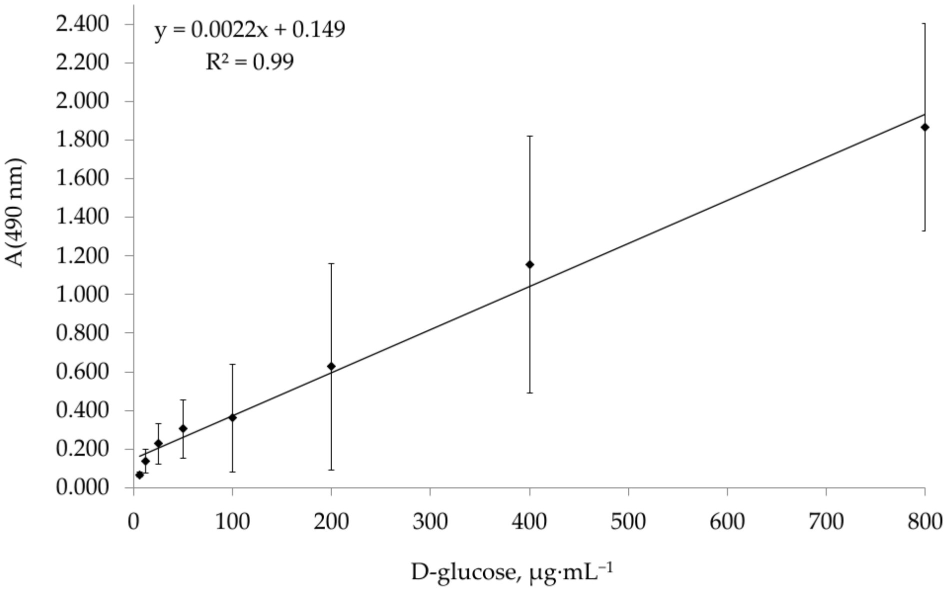

Figure S1. A calibration curve showing the relationship between the $A_{490\ nm}$ absorbance and the concentration of D-glucose in the phenol-sulphuric acid reaction, used for the quantitative analysis of carbohydrates.

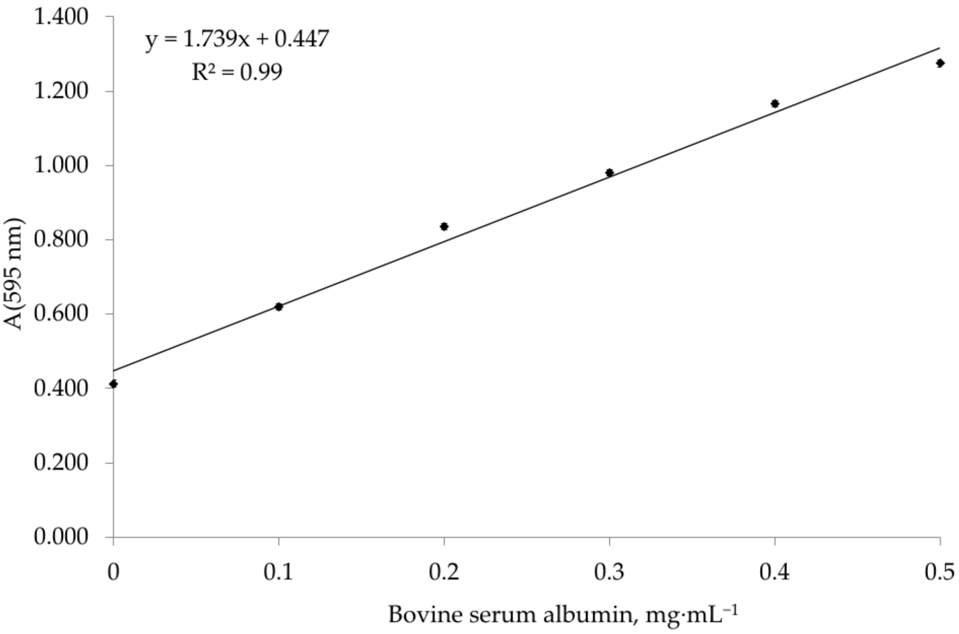

Figure S2. A calibration curve showing the relationship between the $A_{595\ nm}$ absorbance and the concentration of bovine serum albumin in Bradford analysis.

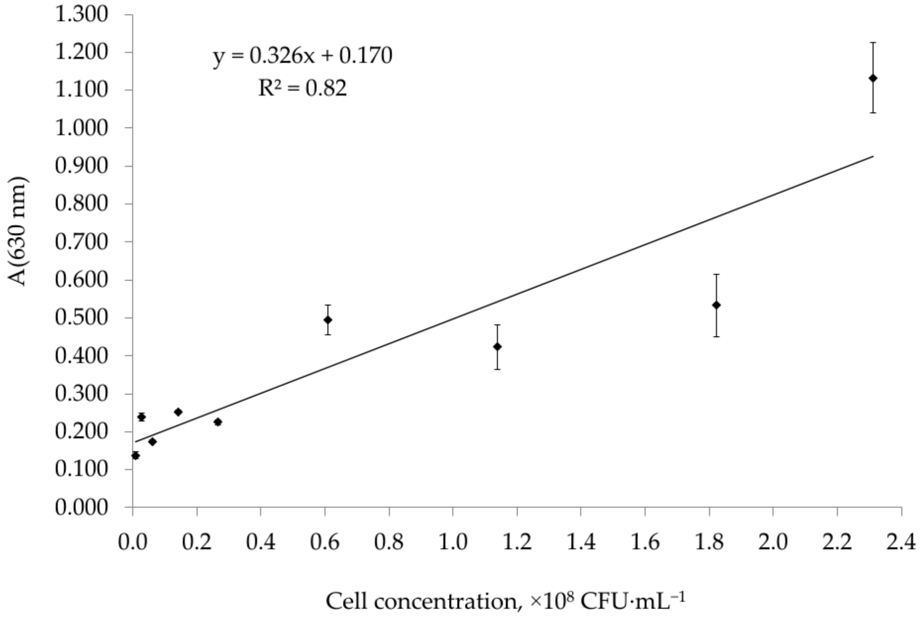

Figure S3. A calibration curve showing the relationship between the $A_{630\ nm}$ absorbance and the cell concentration in the suspension following crystal violet staining.

Table S2. Adhesive activities of *Rhodococcus* spp. towards polystyrene

Strain	Number of adhered cells	
	×10^7 CFU·cm^{-2}	%
R. opacus IEGM 249	4.248 ± 1.379	64
R. rhodochrous IEGM 1162	4.176 ± 1.294	63
R. cerastii IEGM 1327	1.730 ± 0.380	26
R. erythropolis IEGM 788	1.621 ± 0.110	24
R. rhodochorus IEGM 1363	1.477 ± 0.532	22
R. ruber IEGM 1391	1.168 ± 0.323	18
R. rhodochrous IEGM 1360	1.112 ± 0.170	17
R. corynebacterioides IEGM 1202	1.080 ± 0.287	16
R. rhodochrous IEGM 107	0.916 ± 0.377	14
R. wratislaviensis IEGM 1171	0.869 ± 0.130	13
R. cercidiphylli IEGM 1184	0.808 ± 0.296	12
R. aetherivorans IEGM 1367	0.716 ± 0.301	11
R. pyridivorans IEGM 1142	0.525 ± 0.163	8
R. fascians IEGM 1233	0.494 ± 0.179	7
R. ruber IEGM 1122	0.474 ± 0.163	7
R. ruber IEGM 1121	0.376 ± 0.047	6
R. gingshegii IEGM 267	0.301 ± 0.098	5
R. opacus IEGM 262	0.208 ± 0.047	3
R. erythropolis IEGM 1020	0.153 ± 0.029	2
R. aetherivorans IEGM 1250	Below detectable level	Below detectable level
R. erythropolis IEGM 1415	Below detectable level	Below detectable level
R. globerulus IEGM 1203	Below detectable level	Below detectable level
R. jostii IEGM 60	Below detectable level	Below detectable level
R. jostii IEGM 68	Below detectable level	Below detectable level
R. opacus IEGM 2226	Below detectable level	Below detectable level
R. gingshegii IEGM 1348	Below detectable level	Below detectable level
R. gingshegii IEGM 1359	Below detectable level	Below detectable level

Means ± standard deviations are shown.

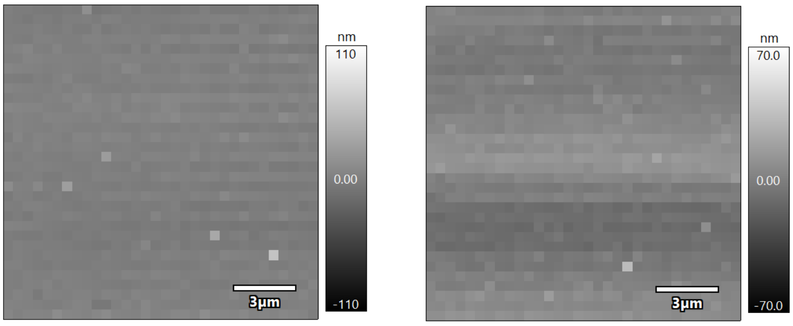

(a)

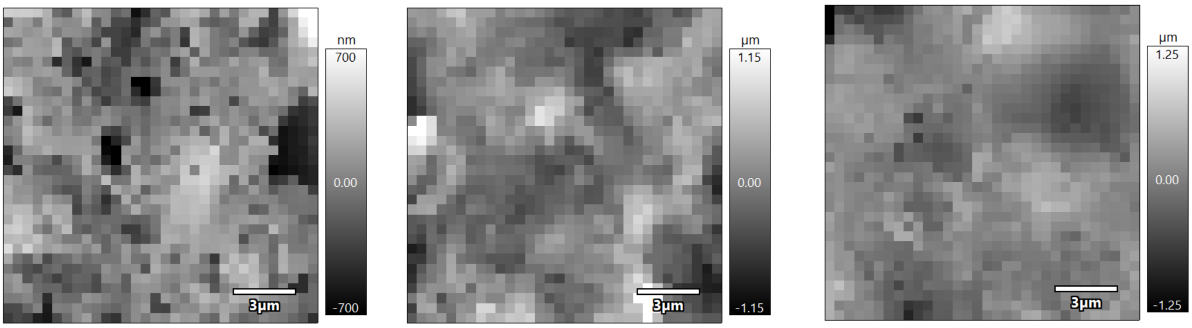

(b)

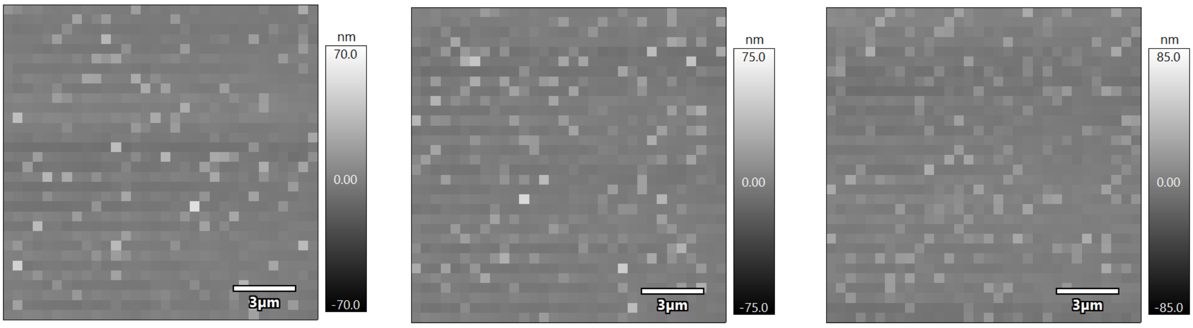

(c)

Figure S4. Topographic maps of an unmodified **(a)** and an EPS-coated **(b)** cover glass surface, scanned with an unmodified cantilever, and an unmodified cover glass surface scanned with a cantilever coated with the EPSs **(c)**. EPSs produced by *R. ruber* IEGM 231 were used. The slope of the topographic maps was eliminated using the Planefit function.

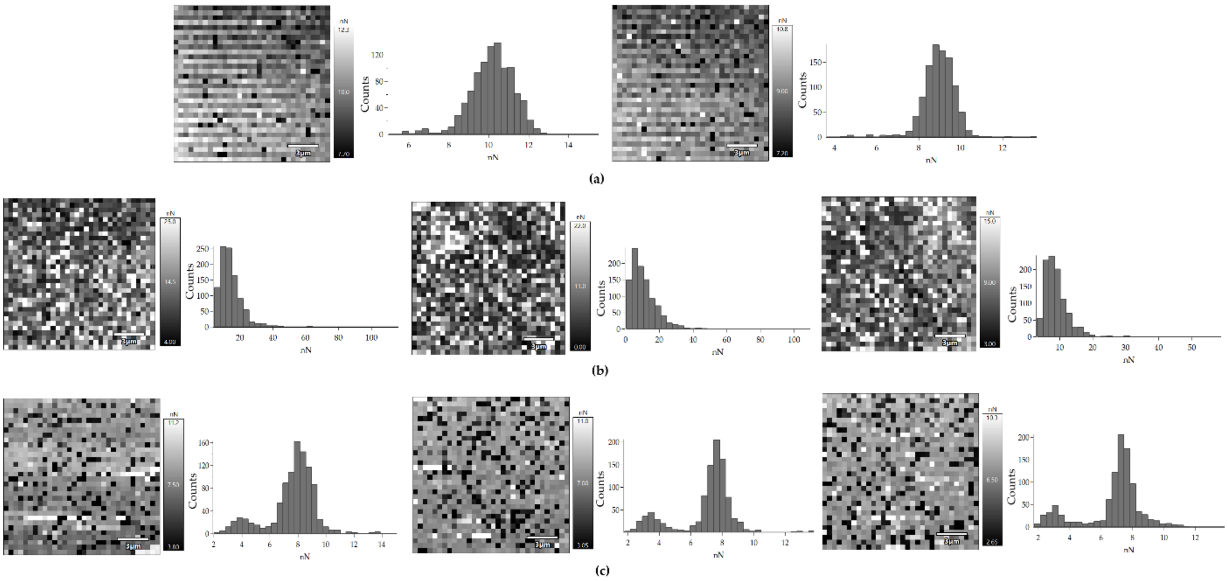

Figure S5. Adhesion force maps and distribution of F_a values for an unmodified **(a)** and an EPS-coated **(b)** cover glass surface, scanned with an unmodified cantilever, and for an unmodified cover glass surface, scanned with a cantilever coated with the EPSs **(c)**. EPSs produced by *R. ruber* IEGM 231 were used.

Table S3. Annotated genes and enzymes that are likely to be involved in the biosynthesis of exopolysaccharides in *Rhodococcus*

Gene	Encoded enzyme(s)	Reaction(s) catalyzed by the encoded enzyme(s)	Number of harboring strains
gtfs	Glycosyltransferases	Activated nucleotide sugar donor (UDP-glucose, UDP-galactose, GDP-mannose, etc.) + acceptor → elongated polysaccharide + nucleotide	38
capA, *pslA*	Capsular polysaccharide biosynthesis proteins	Various enzymes participated in the synthesis of capsule (exopolysaccharides covalently bound to the cell wall) including polymerization, export/translocation, and chain modification processes	30
–	Undecaprenyl-phosphate galactose phosphotransferase (EC 2.7.8.6)	UDP-D-galactose + antigen carrier (phospho)lipid phosphate → UMD + galactosediphosphate antigen carrier (phospho)lipid	30
metX, *metXA*, *cysE*, *dcsE*	Homoserine O-acetyltransferase (EC 2.3.1.31)	Acetyl-CoA + L-homoserine → CoA + O-acetyl-L-homoserine	29
wwbL	N-acetylglucosaminyl-diphospho-decaprenol L-rhamnosyltransferase (EC 2.4.1.289)	dTDP-6-deoxy-beta-L-mannose + N-acetyl-alpha-D-glucosaminyl-diphospho-trans,octacis-decaprenol → dTDP + alpha-L-rhamnopyranosyl-(1->3)-N-acetyl-alpha-D-glucosaminyl-diphospho-trans,octacis-decaprenol	20
plsC	Acyl-CoA:1-acyl-sn-glycerol-3-phosphate acyltransferase (EC 2.3.1.51)	1-acyl-sn-glycero-3-phosphate + acyl-CoA → 1,2-diacyl-sn-glycero-3-phosphate + CoA	1

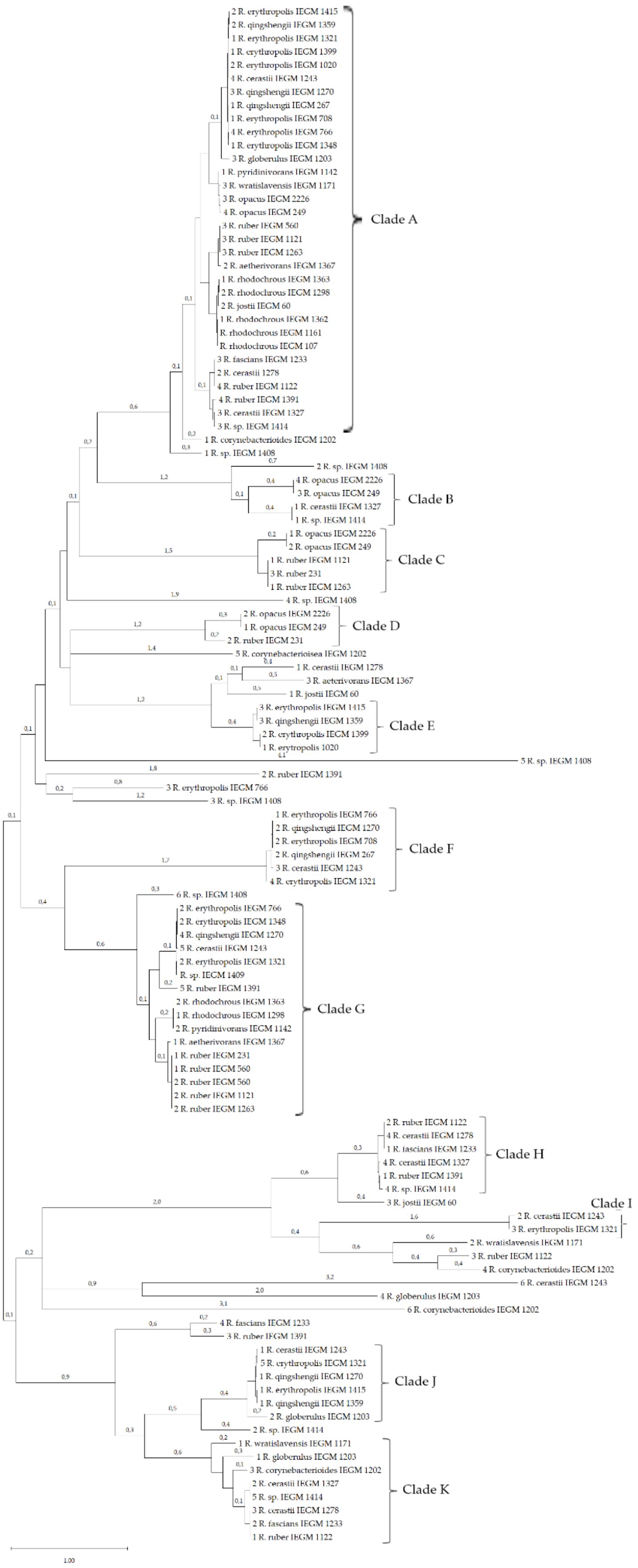

Figure S6. Phylogenetic tree of *Rhodococcus* glycosyltransferases constructed using aligned amino acid sequences. It was created using the neighbor-joining method in MEGA. The tree is rooted at the midpoint. Branch lengths reflect similarities between proteins.

Table S4. Biosynthetic gene clusters probably related to the synthesis of exopolysaccharides in *Rhodococcus* spp.

Strain	Most similar known cluster	Similarity, %	Type	Location
R. aetherivorans IEGM 1367	SF2575 Polyketide: Type II polyketide + Saccharide: Hydrid/tailoring saccharide	6	terpene	Region 11.3 from 677,375 to 698,484 bp
R. cerastii IEGM 1243	acarbose Saccharide	7	PKS-like, amglyccycl	Region 1.2 from 192,618 to 233,646 bp
	SF2575 Polyketide: Type II polyketide + Saccharide: Hydrid/tailoring saccharide	6	NRPS, terpene	Region 6.2 from 242,563 to 296,863 bp
R. cerastii IEGM 1278	SF2575 Polyketide: Type II polyketide + Saccharide: Hydrid/tailoring saccharide	6	terpene	Region 4.3 from 255,278 to 276,420 bp
	hydromycin A Saccharide	6	arylpolyene	Region 5.1 from 209,015 to 250,172 bp
R. cerastii IEGM 1327	SF2575 Polyketide: Type II polyketide + Saccharide: Hydrid/tailoring saccharide	6	terpene	Region 37.1 from 113,094 to 134,239 bp
R. corynebacterioides IEGM 1202	SF2575 Polyketide: Type II polyketide + Saccharide: Hydrid/tailoring saccharide	6	terpene	Region 4.1 from 67,939 to 89,072 bp
R. erythropolis IEGM 708	SF2575 Polyketide: Type II polyketide + Saccharide: Hydrid/tailoring saccharide	6	NRPS, terpene	Region 87.1 from 62,597 to 116,882 bp
R. erythropolis IEGM 766	SF2575 Polyketide: Type II polyketide + Saccharide: Hydrid/tailoring saccharide	6	NRPS, terpene	Region 11.2 from 231,397 to 285,697 bp
R. erythropolis IEGM 788	acarbose Saccharide	7	PKS-like	Region 44.1 from 1 to 13,064 bp
R. erythropolis IEGM 1020	acarbose Saccharide	7	PKS-like, amglyccycl	Region 11.2 from 543,686 to 584,714 bp
	SF2575 Polyketide: Type II polyketide + Saccharide: Hydrid/tailoring saccharide	6	NRPS, terpene	Region 22.2 from 253,395 to 307,725 bp
R. erythropolis IEGM 1321	coelichelin NRP	27	NRPS	Region 197.1 from 134,632 to 201,190 bp
	acarbose Saccharide	7	PKS-like, amglyccycl	Region 175.1 from 524,868 to 559,709 bp
	SF2575 Polyketide: Type II polyketide + Saccharide: Hydrid/tailoring saccharide	6	NRPS, terpene	Region 197.2 from 223,962 to 278,247 bp
R. erythropolis IEGM 1348	SF2575 Polyketide: Type II polyketide + Saccharide: Hydrid/tailoring saccharide	6	NRPS, terpene	Region 53.1 from 52,901 to 107,201 bp

R. globerulus IEGM 1203	SF2575 Polyketide: Type II polyketide + Saccharide: Hydrid/tailoring saccharide	6	NRPS, terpene	Region 28.2 from 102,780 to 156,966 bp
R. opacus IEGM 2226	glycopeptidolipid Saccharide	7	NRPS	Region 33.1 from 46,415 to 130,471 bp
	SF2575 Polyketide: Type II polyketide + Saccharide: Hydrid/tailoring saccharide	6	terpene	Region 44.2 from 262,183 to 283,331 bp
R. opacus IEGM 249	SF2575 Polyketide: Type II polyketide + Saccharide: Hydrid/tailoring saccharide	6	terpene	Region 55.2 from 258,972 to 279,727 bp
R. pyridinivorans IEGM 1142	SF2575 Polyketide: Type II polyketide + Saccharide: Hydrid/tailoring saccharide	6	Terpene, NRPS	Region 22.1 from 328,092 to 398,906 bp
R. qingshengii IEGM 1359	Iomaiviticin A/ Iomaiviticin C/ Iomaiviticin D/ Iomaiviticin E Polyketide: Type II polyketide + Saccharide: Hydrid/tailoring saccharide	3	NRPS	Region 22.1 from 91,889 to 158,699 bp
R. rhodochrous IEGM 107	ectoine Other	75	ectoine	Region 11.1 from 160,355 to 170,753 bp
	SF2575 Polyketide: Type II polyketide + Saccharide: Hydrid/tailoring saccharide	6	terpene	Region 11.2 from 540,164 to 561,279 bp
	kendomycin B Polyketide	6	NRPS	Region 11.3 from 595,670 to 642,906 bp
R. rhodochrous IEGM 1360	SF2575 Polyketide: Type II polyketide + Saccharide: Hydrid/tailoring saccharide	6	terpene	Region 11.2 from 81,884 to 102,999 bp
R. rhodochrous IEGM 1362	SF2575 Polyketide: Type II polyketide + Saccharide: Hydrid/tailoring saccharide	6	terpene	Region 10.2 from 81,980 to 103,095 bp
	prejadomycin / rabelomycin / gaudimycin C / gaudimycin D / UWM6 / gaudimycin A Polyketide: Type II polyketide + Saccharide: Hydrid/tailoring saccharide	4	NRPS	Region 42.1 from 1 to 40,171 bp
R. ruber IEGM 231	SF2575 Polyketide: Type II polyketide + Saccharide: Hydrid/tailoring saccharide	6	terpene	Region 5 from 1,773,262 to 1,794,386 bp
R. ruber IEGM 1121	SF2575 Polyketide: Type II polyketide + Saccharide: Hydrid/tailoring saccharide	6	terpene	Region 6.1 from 82,099 to 103,223 bp
R. ruber IEGM 1391	acarbose Saccharide	7	PKS-like, amglyccycl	Region 31.1 from 160,630 to 201,667 bp

	Coumermycin A1 Saccharide: Hydrid/tailoring saccharide + Other: Aminocoumarin	6	T1PKS	Region 11.1 from 113,831 to 158,939 bp
	SF2575 Polyketide: Type II polyketide + Saccharide: Hydrid/tailoring saccharide	6	terpene	Region 29.1 from 31,455 to 52,590 bp
	K-252a Alkaloid	5	NRPS-like	Region 29.3 from 237,140 to 280,667 bp
R. wratislaviensis IEGM 1171	oxalomycin B NRP + Polyketide	12	NRPS	Region 4.3 from 380,871 to 430,660 bp
	maduramicin Polyketide + Saccharide	7	NRPS	Region 4.2 from 326,445 to 370,842 bp
	SF2575 Polyketide: Type II polyketide + Saccharide: Hydrid/tailoring saccharide	6	terpene	Region 4.1 from 171,976 to 193,124 bp
Rhodococcus sp. IEGM 1408	SF2575 Polyketide: Type II polyketide + Saccharide: Hydrid/tailoring saccharide	6	terpene	Region 22.1 from 83,539 to 104,738 bp
Rhodococcus sp. IEGM 1409	SF2575 Polyketide: Type II polyketide + Saccharide: Hydrid/tailoring saccharide	6	NRPS, terpene	Region 17.1 from 226,442 to 280,697 bp
Rhodococcus sp. IEGM 1414	SF2575 Polyketide: Type II polyketide + Saccharide: Hydrid/tailoring saccharide	6	terpene	Region 40.1 from 131,540 to 152,685 bp
R. aetherivorans IEGM 1250, *R. cercidiphylli* IEGM 1184, *R. erythropolis* IEGM 1399, IEGM 1415, *R. fascians* IEGM 1233, *R. jostii* IEGM 60, IEGM 68, *R. opacus* IEGM 262, *R. rhodochrous* IEGM 1363, *R. qingshengii* IEGM 1270, *R. rhodochrous* IEGM 1161, IEGM 1162, IEGM 1298, *R. ruber* IEGM 560, IEGM 1122, IEGM 1263: EPS-related BGCs are not found				

Abbreviations: amglyccycl – aminoglycoside/aminocyclitol biosynthesis, NRP – non-ribosomal peptide, NRPS - non-ribosomal peptide synthetase, and T1PKS – type I polyketide synthase.
