# Supplementary figures and images for: The Lipid- and Polysaccharide-Rich Extracellular Polymeric Substances of Rhodococcus Support Biofilm Formation and Protection from Toxic Hydrocarbons
Source: Polymers (Basel). 2025 Jul 10;17(14):1912. doi: 10.3390/polym17141912 (PMC12298843; doi:10.3390/polym17141912)

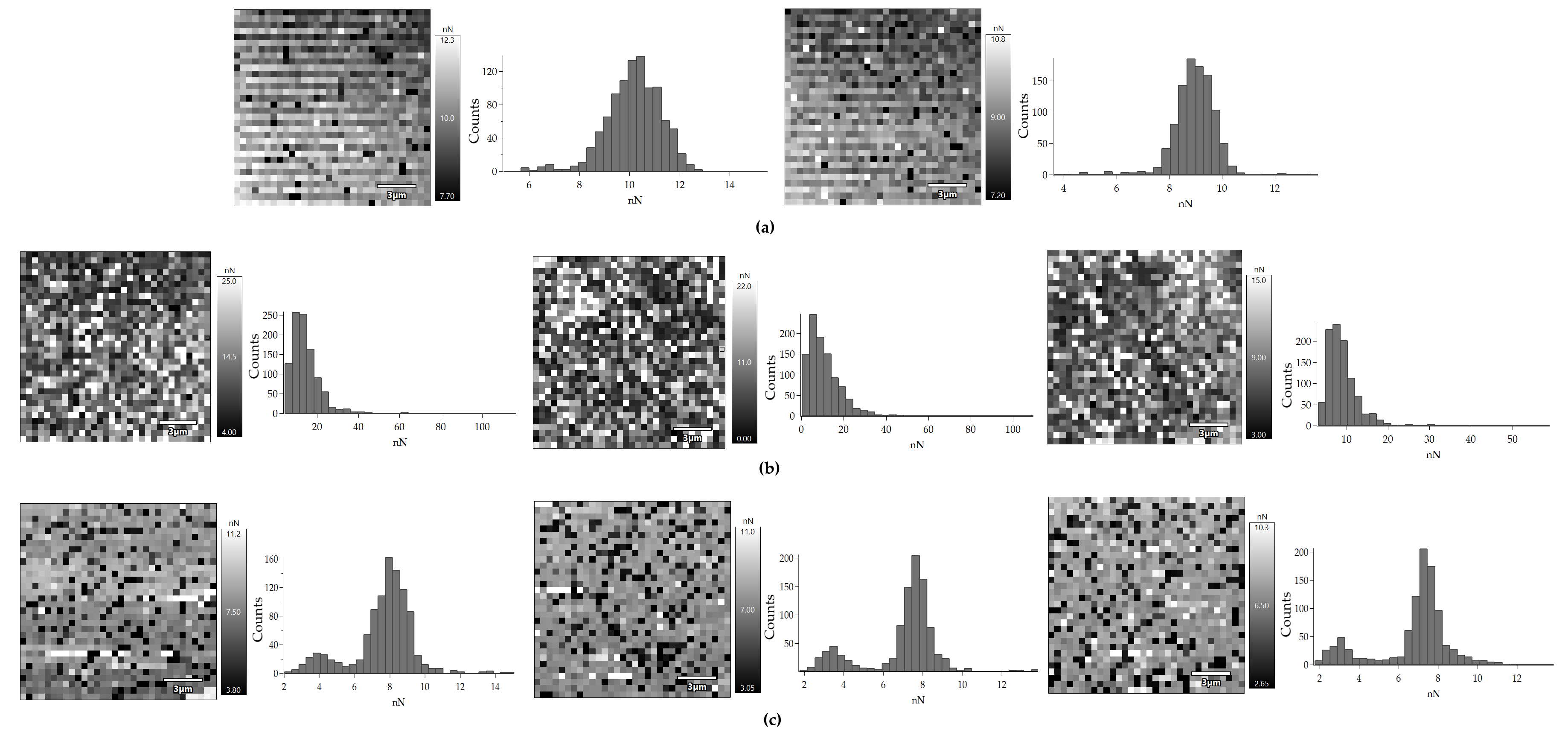

Supplement: Supplementary file 1 [file polymers-17-01912-s001.zip › Figure S5.png]

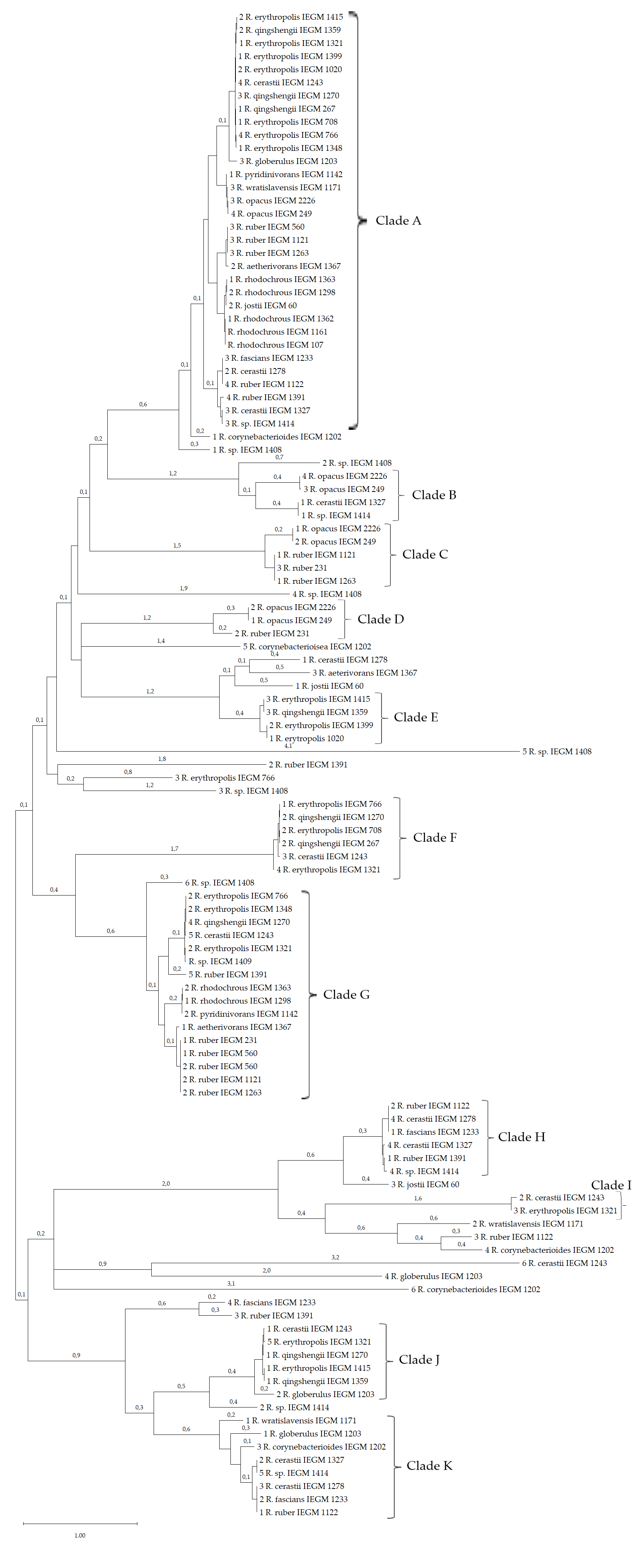

Supplement: Supplementary file 1 [file polymers-17-01912-s001.zip › Figure S6.png]
